# What can urban mobility data reveal about the spatial distribution of infection in a single city?

**DOI:** 10.1186/s12889-019-6968-x

**Published:** 2019-05-29

**Authors:** Robert Moss, Elham Naghizade, Martin Tomko, Nicholas Geard

**Affiliations:** 1 Centre for Epidemiology and Biostatistics, Melbourne School of Population and Global Health, The University of Melbourne, Melbourne, Australia; 2 Department of Infrastructure Engineering, The University of Melbourne, Melbourne, Australia; 3 School of Computing and Information Systems, The University of Melbourne, Melbourne, Australia; 4 The Doherty Institute for Infection and Immunity, Melbourne, Australia

**Keywords:** Urban mobility, Influenza, Spatial epidemiology

## Abstract

**Background:**

Infectious diseases spread through inherently spatial processes. Road and air traffic data have been used to model these processes at national and global scales. At metropolitan scales, however, mobility patterns are fundamentally different and less directly observable. Estimating the spatial distribution of infection has public health utility, but few studies have investigated this at an urban scale. In this study we address the question of whether the use of urban-scale mobility data can improve the prediction of spatial patterns of influenza infection. We compare the use of different sources of urban-scale mobility data, and investigate the impact of other factors relevant to modelling mobility, including mixing within and between regions, and the influence of hub and spoke commuting patterns.

**Methods:**

We used journey-to-work (JTW) data from the Australian 2011 Census, and GPS journey data from the Sygic GPS Navigation & Maps mobile app, to characterise population mixing patterns in a spatially-explicit SEIR (susceptible, exposed, infectious, recovered) meta-population model.

**Results:**

Using the JTW data to train the model leads to an increase in the proportion of infections that arise in central Melbourne, which is indicative of the city’s spoke-and-hub road and public transport networks, and of the commuting patterns reflected in these data. Using the GPS data increased the infections in central Melbourne to a lesser extent than the JTW data, and produced a greater heterogeneity in the middle and outer regions. Despite the limitations of both mobility data sets, the model reproduced some of the characteristics observed in the spatial distribution of reported influenza cases.

**Conclusions:**

Urban mobility data sets can be used to support models that capture spatial heterogeneity in the transmission of infectious diseases at a metropolitan scale. These data should be adjusted to account for relevant urban features, such as highly-connected hubs where the resident population is likely to experience a much lower force of infection that the transient population. In contrast to national and international scales, the relationship between mobility and infection at an urban level is much less apparent, and requires a richer characterisation of population mobility and contact.

**Electronic supplementary material:**

The online version of this article (10.1186/s12889-019-6968-x) contains supplementary material, which is available to authorized users.

## Background

The spread of an infectious disease through a population is an inherently spatial process. Increasingly, mechanistic models of disease transmission seek to incorporate this spatial dimension [1–13]. The spatial distribution of a population is a key determinant of transmission, typically incorporated into a model by representing underlying patterns of population mobility and their implications for contact [4, 6, 12, 14]. Parameterising this mobility behaviour is an ongoing challenge, as available data are often incomplete and biased [15, 16]. Here, we explore and evaluate the use of mobility data and influenza case reports for modelling and predicting spatial patterns of disease at an urban scale.

Non-spatial models of infectious disease models have been successfully used to understand and predict coarse characteristics of disease outbreaks. The spatial heterogenity of populations, however, contributes to the dynamics of spread, and the availability of spatially-indexed mobility and disease data is increasing. Models that incorporate space may therefore provide better explanations for observed epidemiology and more accurate predictions of future outbreaks. Similarly, control interventions may be spatial in nature; for example, the imposition of quarantine zones, or culling of livestock around infected farms [17, 18]. Predictive models that incorporate a spatial dimension are thus important to identify geographic subpopulations at particular risk, and hence to more effectively target preventative measures or allocate public health resources [19].

A key requirement of spatial models is defining how a disease spreads geographically. For human pathogens, this is largely a consequence of population mobility. Determining which types of mobility behaviour are most relevant to disease transmission depends on the geographic scale of interest. Many modelling studies have been concerned with predicting the spread of an infectious disease at a national or international scale. Examples of this include evaluating the effectiveness of various strategies for containing outbreaks of pandemic influenza [2, 20] and Ebola [5]. Of particular interest are the patterns of international movement that can rapidly increase the scale and human cost of disease outbreaks that would once have remained more geographically constrained. Other studies have considered transmission patterns at an urban scale. Again, a key aim is to evaluate the likely effectiveness of spatially defined control measures such as school closures [21]. In the case of dengue, urban-scale spatial models have been used to help explain observed epidemiological patterns, untangling the respective roles of human and vector movement [10, 22]. At a smaller scale still, some studies have focused on disease transmission that may occur within individual buildings such as schools and hospitals [23–27].

We are concerned here with urban scale models that capture the dynamics of disease transmission at the level of a single city. Modern cities represent some of the densest concentrations of human populations ever observed. Larger cities also represent the highly connected hub nodes in global mobility networks. They thus represent ideal settings for incubating and amplifying outbreaks of infectious diseases [28–30]. Urban authorities have responsibility for coordinating public health preparedness and response activities, and detailed models at this scale can provide valuable support for decision making [31].

Spatial models at the urban scale face unique challenges. Population sizes are much smaller than at national and international scales, thus the stochastic nature of infection events will play a stronger role in the dynamics of a disease outbreak. The number of infections at this scale is also likely to be much smaller, therefore the availability of disease surveillance data to calibrate models is often more limited. This issue becomes particularly pronounced when considering spatially-disaggregated data, for example, by suburb or similar statistical unit. For small case counts, the possibility of surveillance data being affected by reporting biases is also increased. Finally, contact and mobility data at the urban scale can be very difficult to observe and accurately infer from data.

In comparison to air and rail travel, or even road traffic between major cities, data on individual patterns of movement at the urban scale is much less readily available. While some data are available on public transport usage, the vast majority of journeys (in Australian cities at least) are by private car. Statistical data is often collected on “journeys to work” (JTW), giving some information on urban mobility patterns. Yet, JTW data only capture a fraction of total journeys made, restricting both the types of journeys captured (i.e., commuting), and the time period captured (census day only). Small-scale studies have measured urban mobility using GPS tracking and/or structured interviews [32, 33]. Larger studies have utilised data generated by mobile phone and social media use [34, 35]. Although these efforts have advanced our understanding of human mobility behaviour, a general solution to parameterising spatial urban-scale models of disease transmission remains an open challenge [8].

The aim of this study is to evaluate the extent to which incorporating urban mobility information into a mathematical model of infection can characterise observed spatial trends in seasonal influenza cases. We examine how the introduction of spatial structure and heterogeneous mixing into a meta-population model of influenza transmission affects the spatial distribution of cases. In this study, we address a particular gap in our understanding: how does the nature of the mobility data, and the spatial scale at which they capture mobility, influence the veracity of the contact model? We therefore explore the impact of different sources of mobility data, and different models of how this mobility translates into patterns of contact. We evaluate how these patterns of contact and infection relate to reported influenza incidence in the city of Melbourne, Australian, given the limitations of these mobility and disease data sets. Finally we discuss how spatial heterogenity in disease risk and reporting may be accounted for in terms of underlying factors, and the implications for improved outbreak forecasting and public health response in the future.

## Methods

### Study area: spatial scale and boundaries

Melbourne is the state capital of Victoria, Australia, and has a population of approximately 4.7 million, making it the second-most populous city in Australia. It is located in south-eastern Australia and has a temperate climate, and so the city experiences seasonal influenza epidemics in the winter months (typically spanning July to October).

The Australian Statistical Geography Standard defines six hierarchical levels, from mesh blocks that typically contain 30–60 dwellings, to whole states and territories. In between these two extremes are four levels of statistical areas: SA1s, SA2s, SA3s, and SA4s. SA2s are the lowest level for which Estimated Resident Population data are available, and typically have populations of 3,000 to 25,000 persons. SA3s typically have populations of 30,000 to 130,000 persons, and respect geographic and socioeconomic similarities, while SA4 regions typically have populations of 300,000 to 500,000 persons and are designed to reflect labour markets [36].

**Fig. 1:**
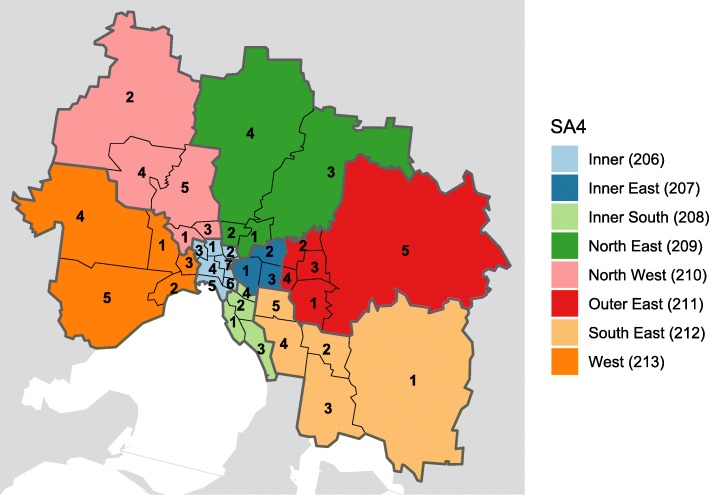
The SA3 and SA4 regions that comprise metropolitan Melbourne. Each SA4 is shown in a different colour, and contains 3–7 SA3s. Each SA3 is identified by a 5-digit number, beginning with the SA4 code (shown in the figure legend) and followed by a number between 1 and *N* (where the SA4 contains *N* SA3s). For example, the central business district (CBD) has the ID 20604; it is located in the Inner (206) SA4, which contains 7 SA3s, and is identified here by the digit “4”

**Fig. 2:**
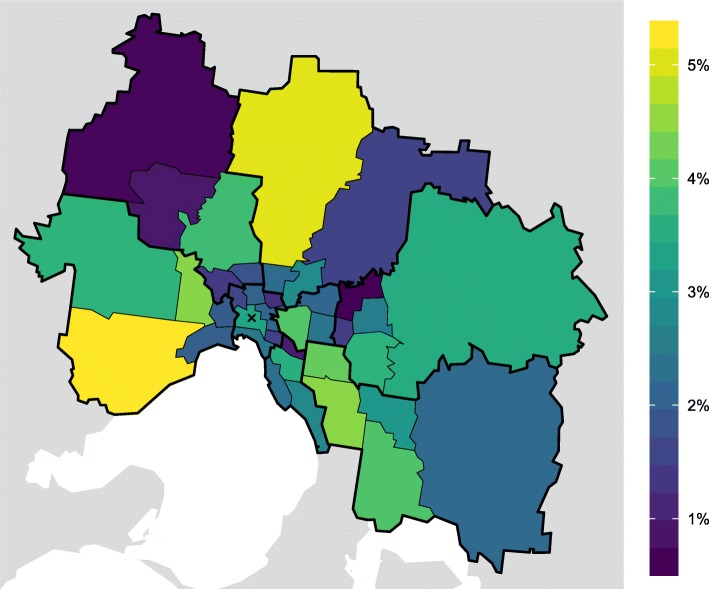
The ABS estimated resident population of each SA3, shown as a percentage of the entire metropolitan population. SA4 boundaries are depicted by thicker lines, and the CBD is identified by the cross

Metropolitan Melbourne comprises 8 SA4s, 38 SA3s, and 296 SA2s. We used SA3s as our spatial analysis unit, representing a compromise between competing objectives: being sufficiently small to characterise spatial variations, and being sufficiently large to have a meaningful number of identified influenza cases in each seasonal epidemic. The division of Melbourne into SA4s and SA3s is shown in Fig. 1, and the variation in resident population is shown in Fig. 2. The estimated resident populations for each SA3 are provided in Additional file 1.

### Urban mobility data sources

The Australian Bureau of Statistics (ABS) has undertaken regular national censuses since 1911, with one held every 5 years since 1961. Here we used “Method of Travel to Work” and “Place of Work” data for SA3 regions from the 2011 census^1^ to characterise urban JTW transport patterns. These data describe the method(s) that *employed individuals* used to travel from their place of usual residence to their place of work on the *day of the census* (Tuesday 9th August). We classified each transport method as being either private transport (e.g., car), public transport (e.g., bus, ferry, train, tram), or other forms of transport (e.g., walking, cycling).

We also obtained origin-destination (OD) data for metropolitan Melbourne in 2016, collected and provided by the “Sygic GPS Navigation & Maps” mobile app^2^. We assumed that this data set only characterised journeys undertaken using private transport, since it is primarily intended for car navigation and is promoted as offering “accurate real-time traffic information”, but the purpose of the trip may vary and is not restricted to commuting.

The ABS census data are publicly available. The Sygic data are not publicly available, and we obtained these data for exclusive academic use.

### Urban mobility mixing matrices

For both data sets we constructed master OD matrices, where each entry represented the *number* of journeys from the origin SA3 (row) to the destination SA3 (column). Matrices were constructed for the following JTW transport modalities: car transport; public transport; and all forms of transport (car, public, and other forms). We separated the JTW data into these modalities so that we could compare the relative effects of public and private transport on the spatial distribution of infection in the model, and also so that we could compare the private transport data to the Sygic GPS data (which is exclusively private transport).

We assumed that these data sets characterised mobility and contact *between regions* but not *within* the region of residence, where the majority of contact is likely to occur in and around the primary residence, which is not captured in these data sets. To keep these two types of contact separate, the diagonal entries (representing journeys that start and end within the same SA3) were set to zero. In order to characterise the relative proportion of journeys from one region to another, we then normalised the matrix rows to sum to unity:

1 $$\begin{array}{*{20}l} F &= \left(\begin{array}{cccc} f_{1,1} & f_{1,2} & \cdots & f_{1,r} \\ f_{2,1} & f_{2,2} & \cdots & f_{2,r} \\ \vdots & \vdots & \ddots & \vdots \\ f_{r,1} & f_{r,2} & \cdots & f_{r,r} \\ \end{array}\right) \end{array} $$

2 $$\begin{array}{*{20}l} f_{i,i} &= 0  \end{array} $$

3 $$\begin{array}{*{20}l} \sum_{j=1}^{r} f_{i,j} &= 1 \quad \forall i \in [1..r]  \end{array} $$

For each data set we constructed a family of mixing matrices *M*, under the assumption that some proportion of infections $\delta _{i}^{H}$ arising from a resident of region *δ*_*i*_ occur in the resident’s home region *i* and that the remaining proportion $\delta _{i}^{*} = 1 - \delta _{i}^{H}$ of infections occur outside of region *i* (4):

4 $$\begin{array}{*{20}l} M &= \left(\begin{array}{llll} \delta_{1}^{H} & \delta_{1}^{*} f_{1,2} & \cdots & \delta_{1}^{*} f_{1,r} \\ \delta_{2}^{*} f_{2,1} & \delta_{2}^{H} & \cdots & \delta_{2}^{*} f_{2,r} \\ \vdots & \vdots & \ddots & \vdots \\ \delta_{r}^{*} f_{r,1} & \delta_{r}^{*} f_{r,2} & \cdots & \delta_{r}^{H} \\ \end{array}\right)  \end{array} $$

### Mixing in the central business district

Melbourne’s Central Business District (CBD, SA3 20604) sits at the centre of hub-and-spoke public transport and road transport networks. It has a resident population of about 148,000 and attracts around *five times as many daily visitors* for a variety of purposes, such as employment, recreation, and tourism. Given this disparity in resident and transient population sizes, we hypothesise visitors to the CBD are much more likely to mix with each other than they are to mix with residents of the CBD. To account for this feature, we also varied the proportion (*δ*_*C*_) of mixing in the CBD that involves the resident population. The rest of the mixing in the CBD, which exclusively involves non-residents, was redistributed to the resident population of each non-CBD region, in proportion to the intensity of mixing between that region and the CBD. Note that this non-resident mixing is entirely different from the effect of $\delta _{i}^{H}$, which distributes an individual’s force of infection across the resident populations of their home SA3 and other SA3s that they visit.

We identify the CBD as region *c*, and calculate the relative mixing that occurs between each non-CBD region and the CBD ($\hat {\mathbf {C}}$), accounting for the resident population *N*_*i*_ in each region *i*:

5 $$\begin{array}{*{20}l} {}\mathbf{C} &= [M_{1,c} \cdot N_{1}, \dots, M_{c-1,c} \cdot N_{c-1}, 0, M_{c+1,c} \cdot N_{c+1}, \dots,\\ &\quad M_{r,c} \cdot N_{r}] \end{array} $$

6 $$\begin{array}{*{20}l} {}\hat{\mathbf{C}} &= \mathbf{C} / \lvert\mathbf{C}\rvert \end{array} $$

Then, assuming that a proportion *δ*_*C*_ of mixing in the CBD involves the resident population and that the remaining proportion $\delta _{C}^{*} = 1 - \delta _{C}$ occurs exclusively between non-residents, we re-distribute some of the mixing between each region *i* and the CBD to the non-CBD regions (including *i*) in proportion to how strongly each region mixes with the CBD:



The three clauses above represent: unadjusted mixing from the CBD to other regions (); reduced mixing from non-CBD region *i* to the CBD (); and increased mixing from non-CBD region *i* to non-CBD region *j* that is mediated by residents from both regions interacting in the CBD (). Note that, just like the unadjusted mixing matrix *M*, each row of this adjusted mixing matrix *M*^′^ sums to unity.

### Mathematical model of infection and spatial interaction

We used an SEIR (susceptible, exposed, infectious, recovered) deterministic compartment model in which the population was divided into 38 patches (1 for each SA3). We assumed that all individuals experienced the same frequency and intensity of interactions, regardless of factors such as age. We also assumed that individuals adhered to their regular mobility patterns irrespective of their current disease state. This assumption is reasonable for infections that are asymptomatic or associated with only mild symptoms. Recent evidence supports a role for asymptomatic cases in the transmission of influenza, showing that aerosol transmission from normal breathing is sufficient for transmission [37], and sneezing and coughing are not required for infection to occur.

The spread of infection between patches was characterised by mixing matrices that were derived from each of the four OD matrices (JTW car transport, JTW public transport, JTW all modalities, and Sygic GPS data). In this study, we ran model simulations for each of 504 different mixing matrices (4 OD matrices, 18 values for $\delta _{i}^{H}$, 7 values for *δ*_*C*_), where each outbreak was started by seeding exposures in a randomly-selected SA3. For each mixing matrix *M*^′^, the daily force of infection vector (*Λ*) was defined as:

8 $$\begin{array}{*{20}l} \Lambda &= \beta \cdot \mathbf{I} \times M^{\prime} \end{array} $$

We recorded the cumulative number of infections in each SA3 (i.e., the epidemic final size) and compared these to the spatially-uniform scenario where infection is distributed uniformly across the whole population. Model parameter values are listed in Table 1, and further details regarding the model are provided in Additional file 1.

**Table 1:** Parameter values for the SEIR model and the mixing matrices

Parameter	Value	
*R*_*o*_	1.4	Basic reproduction number
*σ*	2.0	Inverse of incubation period
*γ*	0.5	Inverse of infectious period
*β*	0.7	Daily force of infection (*R*_0_·*γ*)
*E*_0_	10	Initial number of exposures
$\delta _{i}^{H}: i \in [1..r]$	$\left \{ \frac {1}{20}, \frac {2}{20}, \dots \frac {18}{20}, \frac {19}{20} \right \}$	Fraction of infections that occur in the home region
*δ*_*C*_	$\left \{ \frac {1}{5}, \frac {1}{4}, \frac {1}{3}, \frac {1}{2}, \frac {2}{3}, \frac {3}{4}, \frac {4}{5} \right \}$	Fraction of mixing in CBD that involves residents
*N*_*i*_	varies	Region resident populations

The SA3 resident populations are provided in Additional file 1

### Influenza case notifications data

In the state of Victoria, medical practitioners and laboratories are required to notify the Department of Health and Human Services of cases that meet the Communicable Diseases Network Australia case definition for laboratory-confirmed influenza (one of: detection of virus by nucleic acid testing; isolation of virus by culture; detection of antigen by a validated antigen assay; seroconversion or a fourfold or greater rise in antibody titre to virus). Aggregate notifications data are publicly available, but not for individual SA3s. Permission to use and publish influenza case notifications data for individual SA3s was obtained from the Department of Health and Human Services.

We obtained influenza case notifications data for the 2010–16 influenza seasons in metropolitan Melbourne [38], and allocated each case to an SA3 based on the patient’s postcode of primary residence. Where a postcode defined a region that belonged to multiple SA3s, we allocated fractional cases to each SA3 according to population-weighted correspondences.

The weekly number of influenza cases reported in any SA3 are small (90 cases or fewer, with only 28 occurrences of 50 or more cases in the 38 SA3s over 365 weeks). These time-series are noisy and do not clearly characterise a consistent epidemic, and are thus not appropriate benchmarks against which to evaluate the model output. However, we can estimate the *relative risk of infection* in each SA3 from these data. These relative risks may in turn better reflect the spatial interaction patterns of the population.

The epidemic model characterises *infected individuals*, and the case notifications data characterise *observed disease* (“cases”). Clearly, not all infections will be observed. The relationship between infections and cases is fundamentally complex and uncertain [39], and is affected by factors that may themselves be spatially dependent. The probabilities of exposure, of infection given exposure, and of observed disease given infection are all likely to be spatially dependent. Exposure risk is influenced by mobility patterns and the frequency and intensity of interactions, which are associated with age and location [40, 41]. Similarly, the risk of exposure resulting in infection is influenced by prior experience of disease, vaccination status, and by co-morbidities, all of which are known to vary with geographic location and socioeconomic status [42]. Finally, the probability of being observed given disease will depend in part on an individual’s healthcare seeking behaviour, which has also been observed to vary with socioeconomic status [43].

## Results

**Fig. 3:**
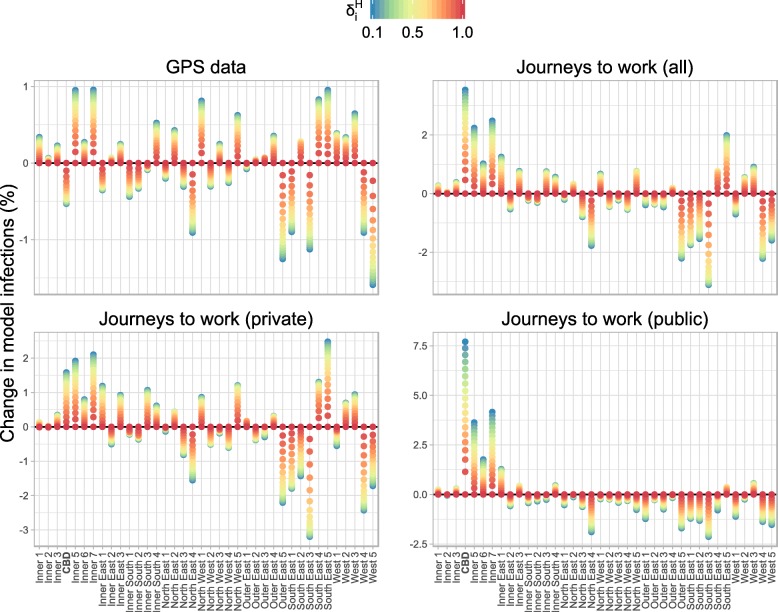
The change in model infections in each SA3 compared to the spatially-uniform model for *δ*_*C*_=0.33, expressed as a percentage of the total number of infections across all SA3s. For the GPS data (top-left) when $\delta _{i}^{h} = 0.1$, an extra 1% of all infections occurred in the each of the Inner 5 (Port Phillip) and Inner 7 (Yarra) SA3s, and about 0.5% fewer of all infections occurred in the CBD. In contrast, for the private journeys to work data (bottom-left) when $\delta _{i}^{h} = 0.1$, an extra 1.5–2% of all infections occurred in each of those SA3s. And for the public journeys to work data (bottom-right) when $\delta _{i}^{h} = 0.1$, these same SA3s experienced an even greater proportion of all infections

**Fig. 4:**
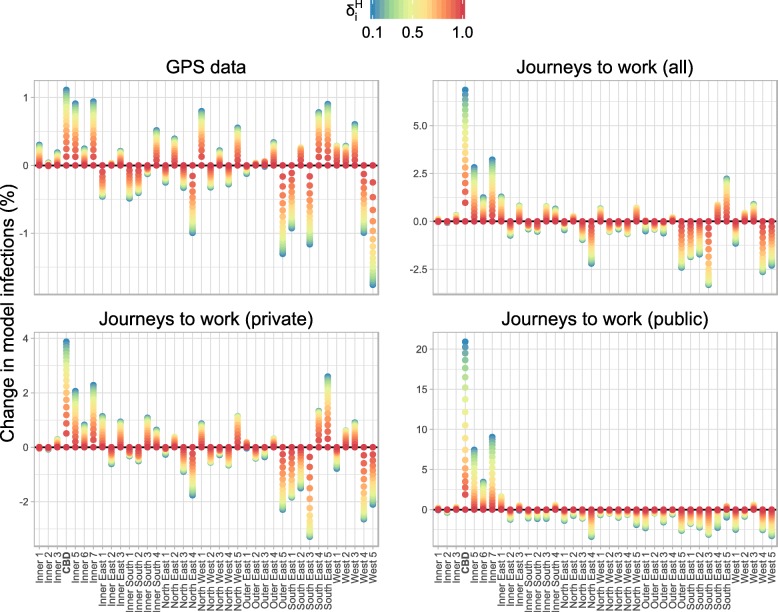
The change in model infections in each SA3 compared to the spatially-uniform model for *δ*_*C*_=0.67, expressed as a percentage of the total number of infections across all SA3s. In comparison to Fig. 3, and as expected, a larger value for *δ*_*c*_ means that a greater proportion of all infections occur in the CBD. In particular, for the GPS data (top-left) the CBD now experiences an *increase* in infections, relative to spatially-uniform model

Introducing spatially-heterogeneous population mixing into the infection model results in a spatially-heterogeneous distribution of infection. The degree of heterogeneity is primarily controlled by the parameter $\delta _{i}^{H}$ — the proportion of infections acquired from an individual that occur in that individual’s home region. This can be seen in Figs. 3 and 4, which show the spatial distribution of infection for each of the OD matrices. Figure 3 shows these distributions for *δ*_*C*_=0.33 (i.e., mixing in the CBD primarily involves visitors) and Fig. 4 shows them for *δ*_*C*_=0.67 (i.e., mixing in the CBD primarily involves CBD residents). As $\delta _{i}^{H} \to 1$ (red points) the dynamics approach that of the non-spatial infection model (and when $\delta _{i}^{H} = 1$ the epidemic would be confined to the region(s) where infections are introduced).

The public transport JTW matrices clearly capture the impact of the spoke-and-hub structure of the public transport network on spatial interactions, with Inner Melbourne (SA4 206) home to an extra 20% of *all infections*. Smaller increases (≈1*%* of all infections) are clustered in the inner east/south: Boroondara (Inner East 1), Whitehorse – West (Inner East 3), Stonnington East (Inner South 4), and Monash (South East 5); and also in the inner west: Maribyrnong (West 3, adjacent to the CBD). The private transport JTW matrices and GPS matrices produce spatial distributions that are (a) similar to each other; and (b) markedly different to those of the public transport JTW matrices. The combined private and public transport JTW matrices also yield results that are near-identical to the GPS matrices, because more than two-thirds of the working population reported that they used private transport to travel to work. This supports our initial assumption about the bias towards private transport in the GPS dataset.

As the value of *δ*_*C*_ increases, so too does the proportion of all infections that occur in the CBD (20604). Relative to the spatially-uniform scenario, this proportion only ever decreases when using the GPS OD matrices and only for small values of *δ*_*C*_ (*δ*_*C*_<0.5). This is apparent in the top-left panel of Fig. 3. For all other mixing matrices (i.e., those using JTW data and/or large values of *δ*_*C*_) there is *always* an elevated proportion of infections in the CBD. This highlights the CBD-centric nature of the JTW data in general, and public transport trips in particular.

**Fig. 5:**
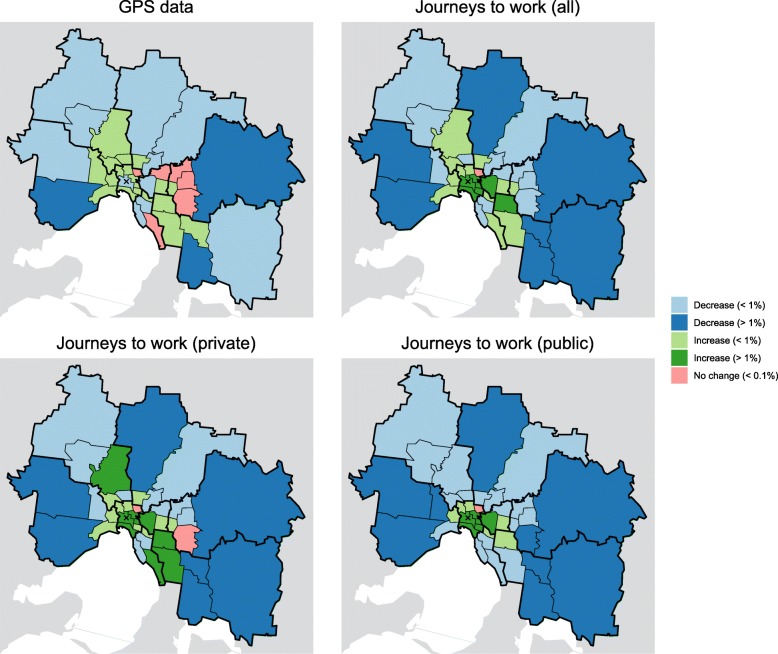
The change in infection distributions, classified by magnitude and direction, for *δ*_*C*_=0.33 and $\delta _{i}^{H} = 0.1$. Results are shown relative to the spatially-uniform scenario. The GPS data and private journeys to work data exhibit similar trends, where increases in disease occur along a north-west to south-east axis, extending beyond the inner-most SA4 regions, and moderate to large decreases in disease in the outer-most regions and inner north-east. The public journeys to work data exhibit a greater concentration of disease in and around the CBD, and also in the inner-east, but without increases in the north-west or south-east

While the results obtained from the public transport JTW matrices are straightforward to interpret — a marked increase in infections in the central and inner east regions — the geographic trends obtained from the other mixing matrices are not immediately evident in Figs. 3 and 4. The results capturing the increase or decrease of the proportion of predicted cases relative to the spatially-uniform scenario by SA3 for each mixing matrix (Fig. 5) show a consistent increase in infections within and around Inner Melbourne (206), and a decrease in the peripheral SA3s in all directions: North East (209), North West (210), Outer East (211), South East (212), and West (213). There are also clear differences between the results from JTW and GPS matrices, particularly in the Inner South (208) and North East (209), and also in the inner regions of the North West (210), Outer East (211), and South East (212). The JTW matrices produce a lower proportion of infections in the Outer North (209) and West (213) than the GPS matrix, which also yielded an increased proportion of infections in the inner north-west.

The division between SA3s with increased and decreased infections is not aligned with the SA4 boundaries shown in Fig. 1. This suggests that SA4 regions are not a suitable higher-level spatial aggregation of metropolitan Melbourne for characterising patterns of urban mobility and their consequences for influenza infection. Instead, the boundary between inner SA4s (206–208) and peripheral outer SA4s (209–213) should be pushed outwards, while retaining the radial division of the outer SA4s.

**Fig. 6:**
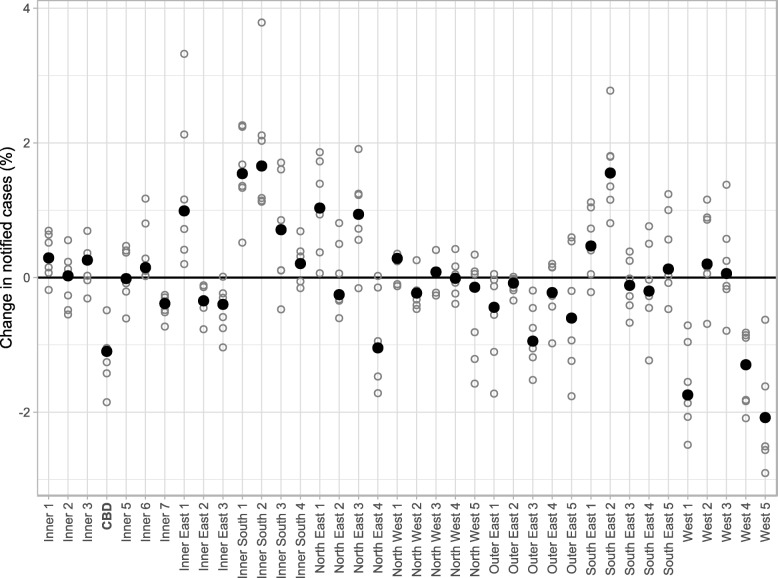
The change in notified influenza cases in the 2010–16 influenza seasons compared to the spatially-uniform model, expressed as a percentage of the total number of cases across all SA3s. Values for each season are shown as hollow points, median values are shown in black. SA3s with greater heterogeneity are more likely to exhibit a consistent increase (alternatively, decrease) in cases for each season

The distribution of influenza cases over metropolitan Melbourne for the 2010–16 influenza seasons is shown in Fig. 6. With the exception of the CBD (20604), the case distribution in Inner Melbourne (206) aligns well with the population distribution. A similar pattern is evident in the Inner East (207), where only Boroondara (20701) has an increased proportion of cases. Most of the Inner South (208) also have an increased proportion of cases, while the North East (209) has increases in Banyule (20901) and Nillumbik - Kinglake (20903) and a decrease in Whittlesea - Wallan (20904). The North West (210) has a near-uniform distribution of cases, and most of the Outer East (211) have a small decrease. The South East (212) has a marked increase in Casey - North (21202), and the West (213) has very large decreases in Brimbank (21301), Melton - Bacchus Marsh (21304), and Wyndham (21305). In comparison to the model results, the distribution of influenza cases exhibits less clustering of SA3s where disease activity is consistently increased or decreased. The blue SA3s (decreased activity) and green SA3s (increased activity) are scattered amongst each other to a greater degree than observed for the model simulations.

**Fig. 7:**
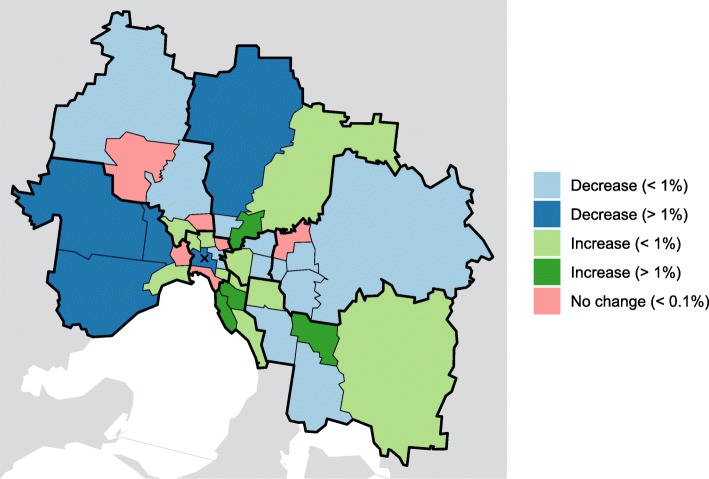
The median spatial distribution of reported influenza cases, relative to the spatially-uniform model. The threshold between small and large changes is 1% of all infections city-wide. In comparison to the model predictions shown in Fig. 5, a greater proportion of cases are observed in the outer east, and there is less clustering of increased cases in and around the CBD
- A small increase in the inner west (West 2);
- Large reductions in the outer west (West 1, West 4, West 5);
- Large reductions in the outer north (North East 4); and
- Large reduction in the outer east in the model simulations, and a small reduction in the influenza cases data in all SA3s except Maroondah (Outer East 3), where the reduction was 0.93% (slightly smaller than the 1% threshold).

Figure 7 shows the spatial variation of the differences between the spatially-uniform scenario and the median annual proportion of notified cases (over the 2010-16 seasons) in each SA3. These variations share a number of similarities with the spatial distributions of infection that are produced by the simulation model for each of the OD data sets (shown in Fig. 5):

- Little spatial heterogeneity across Inner Melbourne (206), except for the CBD;

There are also clear differences between the median distribution of influenza cases and the model outputs:

- An increase in influenza cases in the outer north east (North East 3) and outer south east (South East 1, South East 2), where the JTW models predict a decrease in infections and the GPS model predicts only a very small increase;
- An increase in influenza cases in the inner south, where the model generally predicts a decrease in infections; and
- A decrease in influenza cases in the mid-east, where the JTW models predict an increase in infections and the GPS model predicts no change.

**Fig. 8:**
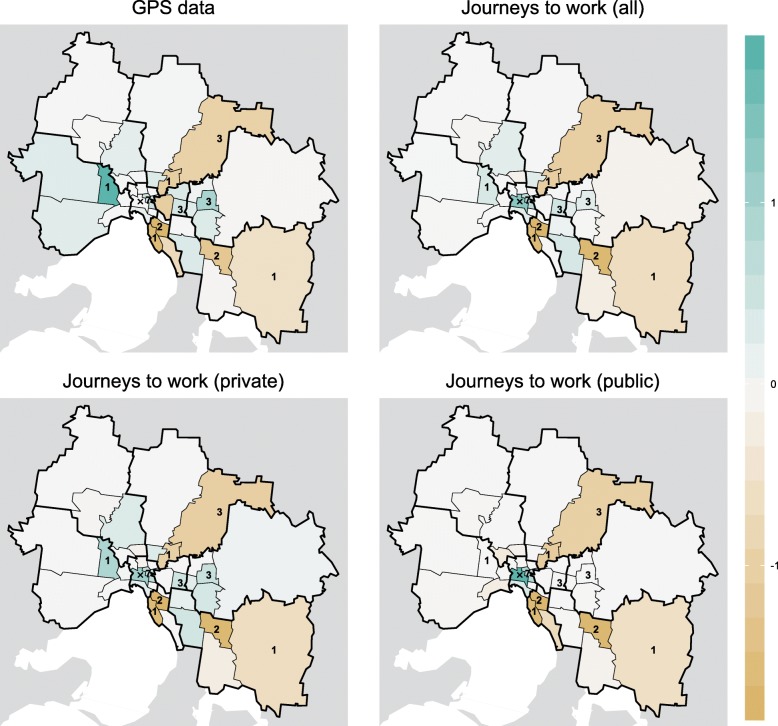
The smallest difference between model infections and influenza cases in each SA3. The scale is the percentage of all infections; positive values mean that the model over-estimates infections, negative values mean that the model under-estimates infections. For 10 of the 38 SA3 regions, the spatially-heterogeneous model predicts the opposite trend (i.e., an increase in infections where there is a decrease in influenza cases, or a decrease in infections where there is an increase in influenza cases) no matter what data set is used. These SA3s are labelled with their identifying digits, as per Fig. 1: Inner 7 (Yarra), Inner East 3 (Whitehorse West), Inner South 1 and 2 (Bayside, Glen Eira), North East 1 and 3 (Banyule, Nillumbik Kinglake), Outer East 3 (Maroondah), South East 1 and 2 (Cardinia, Casey North), and West 1 (Brimbank)

The smallest differences between the distribution of influenza cases and the distribution of model infections, for both the JTW data and the GPS data, are shown in Fig. 8. Here we can see a trend in over-prediction of incidence in the west, north-west, and mid-east, and an under-prediction of incidence in the inner east, inner south-east, outer north-east, and outer south-east.

We also examined the effects of allowing $\delta _{i}^{H}$ to vary between SA3s, by setting it to the proportion of residents in each SA3 who work within their SA3. This had a relatively small effect on the proportion of infections in each SA3, and did not improve the model agreement with the observed distribution of notified influenza cases.

**Fig. 9:**
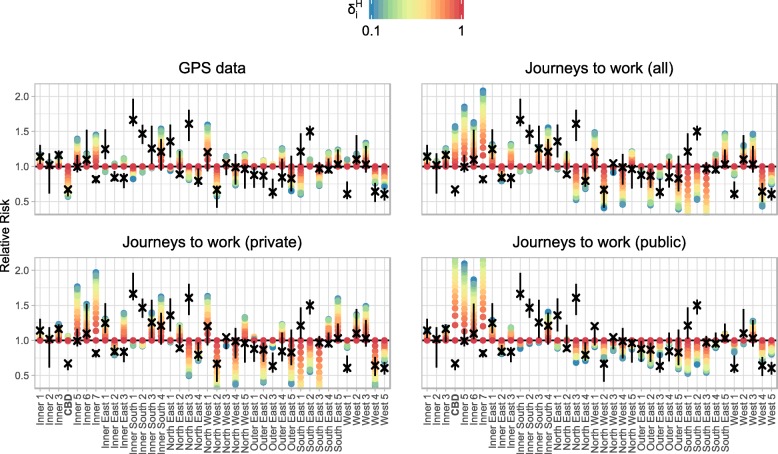
The relative risks of model infection and influenza case notifications, with respect to the spatially-uniform model. Model risks (coloured points) are compared to the influenza case notification risks (black crosses depict median risk, vertical black lines depict interquartile range), for *δ*_*C*_=0.2. The GPS data is the only data set for which we observe a decreased risk of infection in the CBD. Both the GPS data and journeys to work data are capable of yielding relative risks that are similar to those of the influenza case notifications in many SA3s. For 10 of the 38 SA3s, neither data set provides a good match (as identified in Fig. 8)

To account for the near-ten-fold difference in resident populations (ranging from 27,426 to 233,138 in each SA3), we also compared the relative risk of infection in the model to the relative risk of influenza case notifications, as shown in Fig. 9. This figure shows model results for *δ*_*C*_=0.2, which is the region in parameter space where both the absolute and population-weighted differences between the relative risks were smallest. The primary differences are similar to those previously identified: under-estimated risks in the Inner South (208), North East (209), and South East (212), and over-estimated risks in the mid-east (outer regions of 207 and inner regions of 211), and West (213).

## Discussion

### Principal findings

Introducing spatial structure and heterogeneous mixing into the infection model, as informed by Australian Bureau of Statistics journey to work (JTW) data and Sygic GPS data, resulted in a greater proportion of infections occurring both in and around Inner Melbourne (SA4 206), and particularly in the Inner East (207). These are also the most densely-populated regions in metropolitan Melbourne (3,000–5,000 persons per km ^2^). In most of the outer regions, where the population densities are lowest, there was a decreased proportion of infections. Yet, heterogeneous mixing also predicted lower proportion of infections occurring in some of the Inner South (208) and inner regions of the North East (209) and North West (210), where population densities are high. Thus, the spatial heterogeneity in interaction intensities captured by mobility data does not appear to directly reflect population density.

Model results obtained from both the JTW and GPS data sets showed an increase in infections along a north-west to south-east axis, extending from Inner Melbourne (206) into the outer ring of SA4 regions, and also showed a decrease in infections in the outer-most SA3 regions. The influenza case notifications data did not exhibit such a consistent increase in cases from north-west to south-east, while an increase in cases was observed in the Inner South (208) and the some of the outer-east regions.

Comparing the JTW and GPS data sets, the clearest difference is the proportion of all infections that are predicted to occur in the CBD (20604). Models informed by JTW data *always* resulted in a large increase in CBD infections, even when as little as 20% of all mixing in the CBD involved the resident population. This is the consequence of (a) Melbourne’s spoke-and-hub transport network; (b) the concentration of the labour workforce in and around the CBD; and (c) the interaction data capturing exclusively journeys to work. In contrast, the GPS data characterise a greater variety of private transport journeys across the city, with a somewhat lesser — but still substantial — concentration of journeys to the CBD. When the majority of mixing in the CBD did not involve the resident population (i.e., *δ*_*C*_<0.5) there was a *decrease* in CBD infections.

The CBD population is notably under-represented in the influenza case notifications data. While these data are subject to ascertainment biases that render them an unsuitable indicator of the spatial heterogeneity of disease, it is worth noting that (a) other SA3s in Inner Melbourne (206) are not under-represented in these data; and (b) the only mixing matrices able to reproduce this pattern were derived from the GPS data and used small values of *δ*_*C*_. The spatially-explicit SEIR model was able to reproduce some of the features of the case notifications data, particularly in Inner Melbourne (206), the inner west, and many of the outer regions. But there were also clear differences between the model output and the case notifications data in the outer north-east, outer south-east, and Inner South (208). If these urban mobility data sets accurately characterised population mixing *as it pertains to human-to-human infection*, then these differences would be indicative of the ascertainment biases in the case notifications data. But given the richness of population mobility and contact patterns [33, 44] and the limitations of the mobility data sets available (e.g., in terms of the population sample, the limited capture of transport by transport modalities other than personal vehicles, lack of knowledge regarding lengths of stay), this cannot be claimed. Further untangling this relationship between observed mobility and notified disease cases requires a richer characterisation of mobility and interaction in the urban environment, and having a better understanding of “the processes that determine how persons are identified by surveillance systems” [39], such as access to healthcare.

### Study strengths

In this study we investigated the implications of human mobility for the spread of infection at the urban scale. The novelty of this study is two-fold. First, thus far much greater attention has been paid to the role of mobility on infection at either large spatial scales (e.g., national [2, 12, 14, 45, 46] and international [6, 15, 47, 48]) or substantially smaller scales (e.g., individual schools [49] and universities [27]), than the scale of a single city. At large scales, the analyses are less nuanced and hence the bias in the mobility data may be of less importance. Conversely, at very fine scales, rich data at individual levels can be captured. Second, comparatively few studies have combined detailed mobility data with spatially-indexed disease data in order to investigate the spatial risk profile of infection; we are not aware of any studies that have done so at an urban scale. Existing studies have primarily focused on using high-resolution mobility data and individual-based models to estimate or fit the basic reproduction number [10, 21, 50, 51], or have used spatially-indexed disease data to infer “effective” contact patterns [52] (i.e., combining contact and case ascertainment).

We further incorporated domain knowledge about Melbourne’s transport network and the role of the CBD as a central hub with an extremely high number of non-resident visitors and workforce. This characterisation of the CBD is evident in the influenza case data: compared to all of the neighbouring SA3s, the CBD has a markedly lower relative risk of notified influenza cases (Fig. 6). This approach can be extended to other transport and workforce hubs, and can also be generalised to incorporate other forms of domain knowledge about urban mobility that are not explicitly captured in the mobility data. This represents a fundamental shift from treating incoming mobility as exerting a force of infection on the *resident* population, to treating the resident and *transient* populations as distinct groups with disparate risks of infection.

We also identified the misalignment of SA4 boundaries with clusters of increased or decreased infections. Therefore, if a higher level of spatial aggregation than SA3s were desired, the use SA4s as the unit of aggregation would not be appropriate. A better fitting spatial aggregation would retain the radial division of SA4s, but would push the boundary that separates the inner SA4s (206–208) from the outer SA4s (209–213) outwards. This unsuitability is not surprising, as SA4s are designed to reflect labour markets rather than the spread of infection, and therefore focus on the employed workforce. This highlights differences in mobility between urban sub-populations. Consequently, appropriate spatial aggregations are not necessarily well-aligned across different sub-populations and scales.

We targeted seasonal influenza in this study as it produces regular, large-scale epidemics, resulting in a large absolute number of cases in each SA3. The spatial patterns of the infection are thus discernible, even though only a small fraction of the infections are captured by the case notifications. For diseases where even a few cases merit intensive interventions (e.g., measles, smallpox), having a detailed understanding of the relationship between urban mobility and infection is indispensable for targeted localised actions (e.g., contact tracing, school closures, outbreak investigations). Similarly, enhanced surveillance protocols in the event of an influenza pandemic, such as first-few-hundred studies, could also be supported by insights derived from mobility data.

### Study limitations

An inherent challenge in realising this goal is to appropriately identify and account for the limitations of, and biases in, the data capturing mobility. At urban and smaller spatial scales, the stochastic nature of the infection events can dominate the outbreak dynamics and the average mobility patterns of the population may not be strongly reflected in the time-series data for any single outbreak [27]. The mobility patterns at an urban scale are also fundamentally different to those at other scales [53]: they include a mixture of routine journeys (e.g., journey to work or school), work related and logistic journeys, and irregular social trips (e.g., ad-hoc social activities, emergencies). The duration of stay at the journey destination(s) can also vary from minutes to days, and this has different consequences for the risk of infection than in national and international travel, where the minimum duration of stay is likely to be on the order of hours or days. Finally and most importantly these journeys are much less readily *observable* than major highway traffic, airline travel trips, and individual movement in enclosed environments such as schools, shopping malls, and military bases, in particular due to privacy concerns associated with extensive and spatially resolved population tracking [54].

In this study, we characterised urban mixing by origin-destination matrices that captured the frequency of movement from an origin region to a destination region, ignoring the time of day at which these movements occurred. It would be interesting to see how these results compare to a nuanced treatment of time-dependent movement patterns. Such an analysis was not feasible in this study because (a) the journey to work data only characterise the trip *to* work (and do not include journey times); and (b) the GPS data do not include a sufficient sample size at smaller temporal scales, and so we would expect the data to be substantially affected by sampling bias.

Mobile phone tracking data could provide an alternative perspective on urban mobility, by virtue of capturing a broader range of transport modalities, and by representing a much larger (but still biased) sample of the total population due to high mobile ownership rates for adults, adolescents, and even children. However, access to such data is constrained by legislation and would also require data-sharing agreements with individual telecommunications providers. These data are highly sensitive, since they can characterise private features (such as location of residence and daily routines) and are potentially re-identifiable unless provided in a highly aggregated form (such as the OD matrices used in this study).

The alternatives are large datasets (such as those used in this study) that are either spatially and temporally coarse (e.g., JTW), or conversely, with a fine temporal and spatial resolution but covering only a coarse and likely biased sample of the population, (e.g., GPS tracking data such as Sygic). Fine-scale and detailed data have only small coverage, and are compiled by time- and resource-intensive methods, such as computer assisted telephone interviews [33] and contact diaries [44, 55]. Two of these studies [33, 44] were conducted in the Boroondara and Hume local government areas in Melbourne, which *approximately* correspond to (a) Boroondara (Inner East 1); and (b) Sunbury (North West 4) and Tullamarine – Broadmeadows (North West 5) in our study. These detailed studies provide a much richer depiction of individual mobility and social encounter behaviour, capturing heterogeneity by age, gender, type of location and geographic area. In contrast, the JTW and GPS data used in the study reported here comprise substantially larger population samples with a greater geographic coverage, but cannot capture the diversity of mobility and contact experience.

Different surveillance systems for the same population can report different patterns of disease, even after adjusting for the effects of geographical region and age group [56]. This has implications for using surveillance data to characterise and predict disease dynamics [57]. Using spatially-indexed surveillance data to characterise urban disease activity and to detect spatial disease clusters and other patterns is challenging [58]. For this reason, spatially-explicit models of infection at urban scales are of real value, despite the limitations of the available mobility data sets informing the models.

## Conclusions

Human mobility and contact at urban scales are fundamentally more complex and less measurable than at national and international scales, where spatio-temporal patterns of infection are more clearly discerned and for which there exists a range of methods and tools for infectious disease risk assessment. We investigated how two different data sets characterise urban mobility in metropolitan Melbourne, Australia, and the implications mobility has on the spread of infection across the urban system. We have in particular demonstrated how to account for features such as highly-connected regions (hubs) where the resident population is likely to experience a much lower force of infection than the transient population. We have further shown that population mixing models informed by mobility data sets yield spatial distributions of infection that reproduced some of the spatial features of influenza case notifications, despite the inherent limitation of these mobility data and the influenza cases data. A more robust treatment of urban mobility and contact in the context of infectious diseases requires (a) a richer characterisation of population mobility and contact, which are typically only obtainable through intensive small-scale studies; and (b) deeper knowledge of spatial ascertainment biases in the disease surveillance data. The pervasiveness of mobile phone ownership means that mobile phone data could provide a sufficiently-detailed picture of mobility and contact across most demographics. Until available, the relationship between reported disease cases and the population experience of infection remains a fundamental challenge for infectious diseases epidemiology, which new and emerging data sources may help to address [59–61].

## Additional file

Additional file 1 Technical appendix. Details of the model equations, mixing matrix construction, and parameter values. (PDF 111 kb)

## Abbreviations

CBD: Central business district
JTW: Journey to work
OD: Origin-destination
SA1, SA2, SA3, SA4: Statistical area level 1, 2, 3, 4
SEIR: Susceptible –Exposed – Infectious – Recovered

## Footnotes

1
http://abs.gov.au/websitedbs/D3310114.nsf/home/Home

2
http://www.sygic.com

## References

[1] Chowell G, Bettencourt LMA, Johnson N, Alonso WJ, Viboud C (2008). The 1918–1919 influenza pandemic in England and Wales: spatial patterns in transmissibility and mortality impact. Philos Trans R Soc B Biol Sci.

[2] Viboud C, Bjørnstad ON, Smith DL, Simonsen L, Miller MA, Grenfell BT (2006). Synchrony, waves, and spatial hierarchies in the spread of influenza. Science.

[3] Arino J (2017). Spatio-temporal spread of infectious pathogens of humans. Infect Dis Model.

[4] Sattenspiel L, Dietz K (1995). A structured epidemic model incorporating geographic mobility among regions. Math Biosci.

[5] Merler S, Ajelli M, Fumanelli L, Gomes MFC, Piontti APY, Rossi L, Chao DL, Longini IM, Halloran ME, Vespignani A (2015). Spatiotemporal spread of the 2014 outbreak of Ebola virus disease in Liberia and the effectiveness of non-pharmaceutical interventions: a computational modelling analysis. Lancet Infect Dis.

[6] Balcan D, Colizza V, Gonçalves B, Hu H, Ramasco JJ, Vespignani A (2009). Multiscale mobility networks and the spatial spreading of infectious diseases. Proc Natl Acad Sci.

[7] Riley S (2007). Large-scale spatial-transmission models of infectious disease. Science.

[8] Riley S, Eames K, Isham V, Mollison D, Trapman P (2015). Five challenges for spatial epidemic models. Epidemics.

[9] Eggo RM, Cauchemez S, Ferguson NM (2011). Spatial dynamics of the 1918 influenza pandemic in England, Wales and the United States. J Roy Soc Interface.

[10] Karl S, Halder N, Kelso JK, Ritchie SA, Milne GJ (2014). A spatial simulation model for dengue virus infection in urban areas. BMC Infect Dis.

[11] Gog JR, Ballesteros S, Viboud C, Simonsen L, Bjornstad ON, Shaman J, Chao DL, Khan F, Grenfell BT (2014). Spatial transmission of 2009 pandemic influenza in the US. PLOS Comput Biol.

[12] Charu V, Zeger S, Gog J, Bjørnstad ON, Kissler S, Simonsen L, Grenfell BT, Viboud C (2017). Human mobility and the spatial transmission of influenza in the United States. PLOS Comput Biol.

[13] Acevedo MA, Prosper O, Lopiano K, Ruktanonchai N, Caughlin TT, Martcheva M, Osenberg CW, Smith DL (2015). Spatial heterogeneity, host movement and mosquito-borne disease transmission. PLOS ONE.

[14] Charaudeau S, Pakdaman K (2014). Commuter mobility and the spread of infectious diseases: application to influenza in France. PLOS ONE.

[15] Colizza V, Barthélemy M, Barrat A, Vespignani A (2007). Epidemic modeling in complex realities. C R Biologies.

[16] Zhao Z, Shaw S-L, Xu Y, Lu F, Chen J, Yin L (2016). Understanding the bias of call detail records in human mobility research. Int J Geogr Inf Sci.

[17] Tildesley MJ, Savill NJ, Shaw DJ, Deardon R, Brooks SP, Woolhouse MEJ, Grenfell BT, Keeling MJ (2006). Optimal reactive vaccination strategies for a foot-and-mouth outbreak in the UK. Nature.

[18] House T, Baguelin M, Van Hoek AJ, White PJ, Sadique Z, Eames K, Read JM, Hens N, Melegaro A, Edmunds WJ, Keeling MJ (2011). Modelling the impact of local reactive school closures on critical care provision during an influenza pandemic. Proc Roy Soc B Biol Sci.

[19] Wilson JG, Ballou J, Yan C, Fisher-Hoch SP, Reininger B, Gay J, Salinas J, Sanchez P, Salinas Y, Calvillo F, Lopez L, Delima IP, McCormick JB (2010). Utilizing spatiotemporal analysis of influenza-like illness and rapid tests to focus swine-origin influenza virus intervention. Health Place.

[20] Ferguson NM, Cummings DAT, Cauchemez S, Fraser C, Riley S, Meeyai A, Iamsirithaworn S, Burke DS (2005). Strategies for containing an emerging influenza pandemic in southeast asia. Nature.

[21] Milne GJ, Kelso JK, Kelly HA, Huband ST, McVernon J (2008). A small community model for the transmission of infectious diseases: comparison of school closure as an intervention in individual-based models of an influenza pandemic. PLOS ONE.

[22] Stoddard ST, Forshey BM, Morrison AC, Paz-Soldan VA, Vazquez-Prokopec GM, Astete H, Reiner RC, Vilcarromero S, Elder JP, Halsey ES, Kochel TJ, Kitron U, Scott TW (2013). House-to-house human movement drives dengue virus transmission. Proc Natl Acad Sci.

[23] Eames I, Shoaib D, Klettner C, Taban V (2009). Movement of airborne contaminants in a hospital isolation room. J Roy Soc Interface.

[24] Chowell G, Nishiura H, Viboud C (2012). Modeling rapidly disseminating infectious disease during mass gatherings. BMC Med.

[25] Ridenhour BJ, Braun A, Teyrasse T, Goldsman D (2011). Controlling the spread of disease in schools. PLOS ONE.

[26] Goscé L, Barton DAW, Johansson A (2014). Analytical modelling of the spread of disease in confined and crowded spaces. Sci Rep.

[27] Holmes EC, Ghedin E, Halpin RA, Stockwell TB, Zhang X. -Q., Fleming R, Davey R, Benson CA, Mehta S, Taplitz R (2011). Extensive geographical mixing of 2009 Human H1N1 influenza A virus in a single university community. J Virol.

[28] Ali SH, Keil R (2006). Global cities and the spread of infectious disease: the case of severe acute respiratory syndrome (SARS) in Toronto, Canada. Urban Stud.

[29] Balcan D, Gonçalves B, Hu H, Ramasco JJ, Colizza V, Vespignani A (2010). Modeling the spatial spread of infectious diseases: The GLobal Epidemic and Mobility computational model. J Comput Sci.

[30] Neiderud C-J (2015). How urbanization affects the epidemiology of emerging infectious diseases. Inf Ecol Epidemiol.

[31] Bell DM, Weisfuse IB, Hernandez-Avila M, del Rio C, Bustamante X, Rodier G (2009). Pandemic Influenza as 21st Century Urban Public Health Crisis. Emerg Infect Dis.

[32] Vazquez-Prokopec GM, Bisanzio D, Stoddard ST, Paz-Soldan V, Morrison AC, Elder JP, Ramirez-Paredes J, Halsey ES, Kochel TJ, Scott TW, Kitron U (2013). Using GPS Technology to Quantify Human Mobility, Dynamic Contacts and Infectious Disease Dynamics in a Resource-Poor Urban Environment. PLoS ONE.

[33] Rolls DA, Geard NL, Warr DJ, Nathan PM, Robins GL, Pattison PE, McCaw JM, McVernon J (2015). Social encounter profiles of greater Melbourne residents, by location–a telephone survey. BMC Infect Dis.

[34] Noulas A, Scellato S, Lambiotte R, Pontil M, Mascolo C (2012). A Tale of Many Cities: Universal Patterns in Human Urban Mobility. PLoS ONE.

[35] Jiang S, Yang Y, Gupta S, Veneziano D, Athavale S, González MC (2016). The TimeGeo modeling framework for urban mobility without travel surveys. Proc Natl Acad Sci.

[36] Australian Bureau of Statistics. Australian Statistical Geography Standard (ASGS) Volume 1 - Main Structure and Greater Capital City Statistical Areas. Cat. no. 1270.0.55.001. 2011. https://www.abs.gov.au/AUSSTATS/abs@.nsf/DetailsPage/1270.0.55.001July٪202011. Accessed 24 May 2019.

[37] Yan Jing, Grantham Michael, Pantelic Jovan, Bueno de Mesquita P. Jacob, Albert Barbara, Liu Fengjie, Ehrman Sheryl, Milton Donald K. (2018). Infectious virus in exhaled breath of symptomatic seasonal influenza cases from a college community. Proceedings of the National Academy of Sciences.

[38] Moss R, Fielding JE, Franklin LJ, Stephens N, McVernon J, Dawson P, McCaw. JM (2018). Epidemic forecasts as a tool for public health: interpretation and (re)calibration. Aust N Z J Public Health.

[39] Reed C, Chaves SS, Daily Kirley P, Emerson R, Aragon D, Hancock EB, Butler L, Baumbach J, Hollick G, Bennett NM (2015). Estimating influenza disease burden from population-based surveillance data in the United States. PLOS ONE.

[40] Cauchemez S, Valleron A-J, Boëlle P-Y, Flahault A, Ferguson NM (2008). Estimating the impact of school closure on influenza transmission from Sentinel data. Nature.

[41] Mossong J, Hens N, Jit M, Beutels P, Auranen K, Mikolajczyk R, Massari M, Salmaso S, Tomba GS, Wallinga J, Heijne J, Sadkowska-Todys M, Rosinska M, Edmunds WJ (2008). Social contacts and mixing patterns relevant to the spread of infectious diseases. PLOS Med.

[42] Nagata JM, Hernández-Ramos I, Kurup AS, Albrecht D, Vivas-Torrealba C, Franco-Paredes C (2013). Social determinants of health and seasonal influenza vaccination in adults ≥65 years: a systematic review of qualitative and quantitative data. BMC Public Health.

[43] Haroon SMM, Barbosa GP, Saunders PJ (2011). The determinants of health-seeking behaviour during the A/H1N1 influenza pandemic: an ecological study. J Public Health.

[44] Campbell PT, McVernon J, Shrestha N, Nathan PM, Geard N (2017). Who’s holding the baby? A prospective diary study of the contact patterns of mothers with an infant. BMC Infect Dis.

[45] Ajelli M, Gonçalves B, Balcan D, Colizza V, Hu H, Ramasco JJ, Merler S, Vespignani A (2010). Comparing large-scale computational approaches to epidemic modeling: agent-based versus structured metapopulation models. BMC Infect Dis.

[46] Geoghegan JL, Saavedra AF, Duchêne S, Sullivan S, Barr I, Holmes EC (2018). Continental synchronicity of human influenza virus epidemics despite climactic variation. PLOS Pathog.

[47] Tizzoni M, Bajardi P, Poletto C, Ramasco JJ, Balcan D, Gonçalves B, Perra N, Colizza V, Vespignani A (2012). Real-time numerical forecast of global epidemic spreading: case study of 2009 A/H1N1pdm,. BMC Med.

[48] Tizzoni M, Bajardi P, Decuyper A, Kon Kam King G, Schneider CM, Blondel V, Smoreda Z, González MC, Colizza V (2014). On the use of human mobility proxies for modeling epidemics. PLOS Comput Biol.

[49] Cauchemez S, Bhattarai A, Marchbanks TL, Fagan RP, Ostroff S, Ferguson NM, Swerdlow D, working group PH (2011). Role of social networks in shaping disease transmission during a community outbreak of 2009 H1N1 pandemic influenza. Proc Natl Acad Sci.

[50] Eubank S, Guclu H, Kumar VSA, Marathe MV, Srinivasan A, Toroczkai Z, Wang N (2004). Modelling disease outbreaks in realistic urban social networks. Nature.

[51] Soriano-Paños D, Lotero L, Gómez-Gardeñes J, Arenas A. A framework for epidemic spreading in multiplex networks of metapopulations. arXiv e-prints. 2018:1802–039691. 1802.03969v1.

[52] Yang W, Olson DR, Shaman J (2016). Forecasting influenza outbreaks in boroughs and neighborhoods of New York City. PLOS Comput Biol.

[53] Brockmann D, Hufnagel L, Geisel T (2006). The scaling laws of human travel. Nature.

[54] de Montjoye Y-A, Hidalgo CA, Verleysen M, Blondel VD (2013). Unique in the Crowd: The privacy bounds of human mobility. Sci Rep.

[55] Kwok KO, Cowling B, Wei V, Riley S, Read JM (2018). Temporal variation of human encounters and the number of locations in which they occur: a longitudinal study of Hong Kong residents. J Roy Soc Interface.

[56] Thomas EG, McCaw JM, Kelly HA, Grant KA, McVernon J (2015). Quantifying differences in the epidemic curves from three influenza surveillance systems: a nonlinear regression analysis. Epidemiol Infect.

[57] Moss R, Zarebski A, Dawson P, McCaw JM (2017). Retrospective forecasting of the 2010–14 Melbourne influenza seasons using multiple surveillance systems. Epidemiol Infect.

[58] Mathes RW, Lall R, Levin-Rector A, Sell J, Paladini M, Konty KJ, Olson D, Weiss D (2017). Evaluating and implementing temporal, spatial, and spatio-temporal methods for outbreak detection in a local syndromic surveillance system. PLOS ONE.

[59] Bettencourt LM, Ribeiro RM, Chowell G, Lant T, Castillo-Chavez C. Intelligence and Security Informatics: Biosurveillance In: Zeng D, Gotham I, Komatsu K, Lynch C, Thurmond M, Madigan D, Lober B, Kvach J, Chen H, editors.. Springer: 2007. p 79–90. 10.1007/978-3-540-72608-1_8.

[60] Althouse BM, Scarpino SV, Meyers LA, Ayers JW, Bargsten M, Baumbach J, Brownstein JS, Castro L, Clapham H, Cummings DA (2015). Enhancing disease surveillance with novel data streams: challenges and opportunities. EPJ Data Sci.

[61] Simonsen L, Gog JR, Olson D, Viboud C (2016). Infectious disease surveillance in the big data era: Towards faster and locally relevant systems. J Infect Dis.

