# Supplementary material for: What can urban mobility data reveal about the spatial distribution of infection in a single city?
Source: BMC Public Health. 2019 May 29;19:656. doi: 10.1186/s12889-019-6968-x (PMC6542035; doi:10.1186/s12889-019-6968-x)
Supplement: Supplementary file 1 — Technical appendix. Details of the model equations, mixing matrix construction, and parameter values. (PDF 111 kb) [file 12889_2019_6968_MOESM1_ESM.pdf]

The spatial infection model

We assume that infection is acquired from individuals who are either asymptomatic or who have only mild symptoms, and who are therefore following their regular mobility patterns. In other words, the spatial distribution of infection results from the routine mobility patterns of susceptible and infectious individuals rather than, say, from susceptible individuals visiting non-mobile infectious individuals. We also assume that all individuals experience the same frequency and intensity of interactions, regardless of factors such as age.

While it is untrue that all infections are acquired from individuals with mild symptoms or no symptoms at all, recent evidence shows that influenza transmission can arise via aerosol transmission from normal breathing [1]—i.e., sneezing and coughing are not required for infection to occur. This highlights the role that mildly-symptomatic and asymptomatic individuals can play in the population experience of infection, especially since they are likely to adhere to their regular routines and their routine hygiene practices.

We define the force of infection in region i that is exerted by infectious individuals who reside in region j (β_{ij}), the daily force of infection in region i (Λ_i), and the daily force of infection vector (Λ):

$$\beta_{ij} = \beta \cdot I_j \cdot M'_{j,i} \quad (1)$$

$$\Lambda_i = \sum_{j=1}^{r} \beta_{ij} \quad (2)$$

$$\Lambda = \beta \cdot \mathbf{I} \times M' \quad (3)$$

We then define a meta-population SEIR compartment model of infection with a mean latent period of $1/\sigma$ and a mean infectious period of $1/\gamma$:

$$\frac{dS_i}{dt} = -\Lambda_i \cdot \frac{S_i}{N_i} \quad (4)$$

$$\frac{dE_i}{dt} = \Lambda_i \cdot \frac{S_i}{N_i} - \sigma E_I \quad (5)$$

$$\frac{dI_i}{dt} = \sigma E_I - \gamma I_i \quad (6)$$

$$\frac{dR_i}{dt} = \gamma I_i \quad (7)$$

We define the initial state to be fully-susceptible resident populations in each region, and introduce E_0 exposed individuals into a randomly selection region α:

$$\alpha \sim \mathcal{U}\{1, r\} \tag{8}$$

$$S_i(0) = \begin{cases} N_i & \text{if } i \neq \alpha \\ N_i - E_0 & \text{if } i = \alpha \end{cases} \tag{9}$$

$$E_i(0) = \begin{cases} 0 & \text{if } i \neq \alpha \\ E_0 & \text{if } i = \alpha \end{cases} \tag{10}$$

$$I_i(0) = R_i(0) = 0 \tag{11}$$

The absorbing states all satisfy the following condition:

$$\mathbf{E} = \mathbf{I} = 0 \tag{12}$$

In this model, infectious individuals move around the urban environment and due to their mixing patterns the force of infection can differ between regions:

$$\Lambda_i = \beta \left[I_i \cdot \delta_i^H + \sum_{j \neq i} I_j \cdot \delta_j^* \cdot M'_{j,i} \right] \implies \Lambda_i \not\equiv \Lambda_j$$

Accordingly, there is no simple expression for the epidemic final size in each patch [2, 3].

Parameter values

Parameter	Value	
R_o	1.4	Basic reproduction number
σ	2.0	Inverse of incubation period
γ	0.5	Inverse of infectious period
β	0.7	Daily force of infection ($R_0 \cdot \gamma$)
E_0	10	Initial number of exposures
$\delta_i^H : i \in [1..r]$	$\left\{\frac{1}{20}, \frac{2}{20}, \dots \frac{18}{20}, \frac{19}{20}\right\}$	Fraction of infections that occur in the home region
δ_C	$\left\{\frac{1}{5}, \frac{1}{4}, \frac{1}{3}, \frac{1}{2}, \frac{2}{3}, \frac{3}{4}, \frac{4}{5}\right\}$	Fraction of mixing in CBD that involves residents
N_i	varies	Region resident populations

Table 1: Model parameter values.

SA3	Resident Population
20601	91653
20602	55271
20603	70367
20604	148041
20605	108569
20606	67798
20607	94234
20701	177361
20702	95593
20703	107256
20801	102737
20802	157014
20803	122522
20804	43808
20901	127693
20902	99751
20903	68270
20904	224581
21001	61472
21002	30739
21003	79587
21004	40978
21005	169296
21101	160712
21102	27426
21103	114524
21104	63597
21105	154495
21201	97790
21202	137519
21203	176002
21204	197453
21205	184848
21301	196858
21302	88342
21303	87355
21304	160293
21305	233138

Table 2: The estimated resident population (ERP) for each SA3 in metropolitan Melbourne, 2016, as reported in ABS catalogue number 3218.0: "Regional Population Growth, Australia, 2016".

References

[1] Yan J, Grantham M, Pantelic J, Bueno de Mesquita PJ, Albert B, Liu F, et al. Infectious virus in exhaled breath of symptomatic seasonal influenza cases from a college community. Proceedings of the National Academy of Sciences. 2018 Jan;.

[2] Ma J, Earn DJD. Generality of the Final Size Formula for an Epidemic of a Newly Invading Infectious Disease. Bulletin of Mathematical Biology. 2006 Apr;68(3):679–702. Available from: `http://dx.doi.org/10.1007/s11538-005-9047-7`.

[3] Miller JC. A note on the derivation of epidemic final sizes. Bulletin of Mathematical Biology. 2012 Sep;74:2125–2141.
